# Small Area Estimation of Subdistrict Diabetes Prevalence in the US Virgin Islands, 2021–2022

**DOI:** 10.5888/pcd21.240205

**Published:** 2024-11-07

**Authors:** Katie Labgold, John Orr, Lyña Fredericks, David Delgado, Joseph Roth, Esther M. Ellis

**Affiliations:** 1 Epidemic Intelligence Service, Division of Workforce Development, Centers for Disease Control and Prevention, Atlanta, Georgia; 2 US Virgin Islands Department of Health, Christiansted, Virgin Islands; 3 Office of Readiness and Response, Centers for Disease Control and Prevention, Atlanta, Georgia

**Figure:**
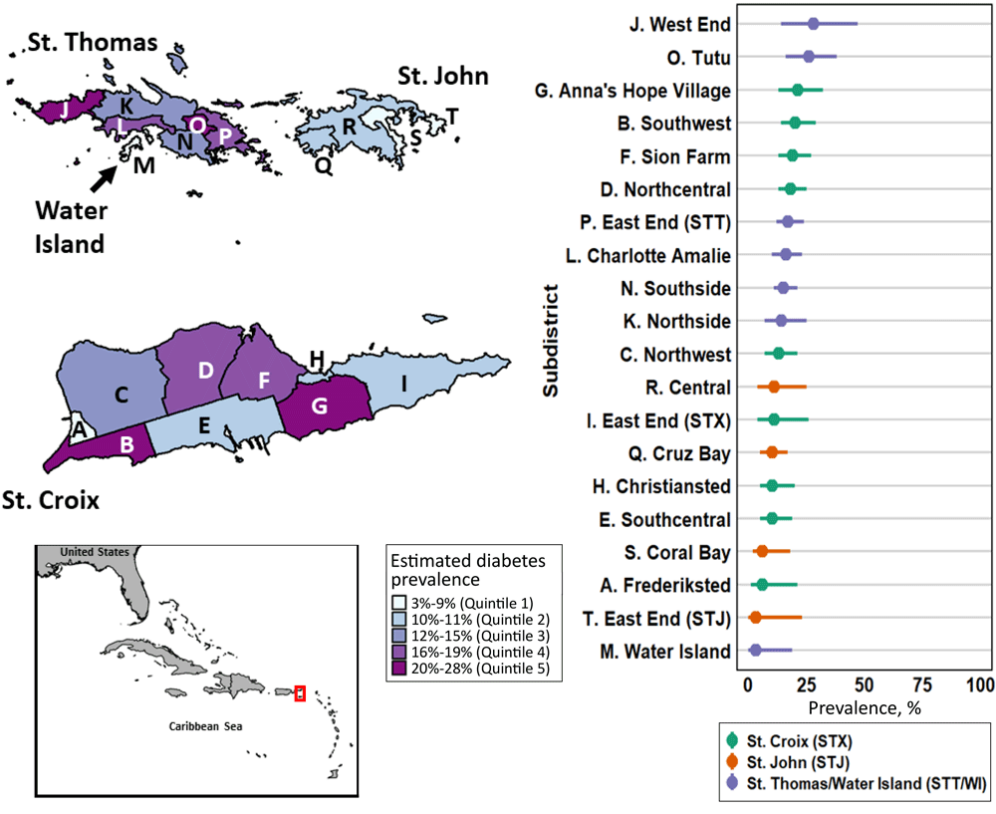
2021–2022 US Virgin Islands (USVI) small area estimates of diabetes prevalence, by subdistrict and quintile class break, for residents aged 18 years or older. The inset map of the Southeastern US and Caribbean indicates the location of USVI. Sources: 2020 United States Decennial Census (1); 2021–2022 Behavioral Risk Factor Surveillance System (2).

## Background

The United States Virgin Islands (USVI) is a US territory located in the Caribbean Sea with 4 inhabited islands that are grouped into 3 districts (ie, county equivalents): St. Croix (41,004 residents; 9 subdistricts), St. John (3,881 residents; 4 subdistricts), and St. Thomas and Water Island (42,261 residents; 7 subdistricts) (1).

USVI obtains territory- and district-level estimates of diabetes prevalence from the Behavioral Risk Factor Surveillance System (BRFSS) (2). These estimates provide an overall understanding of the diabetes burden, which aids in advocacy for diabetes funding and services (eg, policy and grant applications). However, these estimates may fail to capture within-district variation because of the modifiable areal unit problem (MAUP) (3). Estimates below the district level can facilitate public health decision making about programmatic interventions, including using community health workers to support high-prevalence subdistricts by facilitating education and connection to existing diabetes prevention and management programs.

This analysis estimated USVI subdistrict-level diabetes prevalence using small area estimation (SAE). SAE is a technique that leverages multiple population and health data sources available at multiple geographic units (eg, territory, district, subdistrict) to estimate prevalence of health outcomes at a geographic level relevant for program planning (4,5). In USVI, subdistricts are advantageous because they provide a smaller geographic level below the district that is consistent with our understanding of the geographic distribution of disease. Additionally, subdistricts are an appropriate geographic size for prioritizing communities for public health interventions while balancing population size compared with more unstable estate (ie, neighborhood-level) units.

## Data and Methods

This analysis was an ecologic study design that used 2 data sources. Population demographics came from the 2020 Decennial Census (1). USVI receives updated population demographic counts every 10 years. This is USVI’s only population demographic data source with multiple demographic variables (eg, age, sex, race and ethnicity, poverty). The 2020 Census reported 70,060 adult residents.

Diabetes data were obtained from the 2021–2022 USVI BRFSS (2). BRFSS is a nationwide telephone survey with a methodologically intensive participant sampling scheme, accounting for landline and cellular telephone use. Participant responses are weighted to provide generalizable estimates of the total and district-specific populations. Participants were eligible for inclusion if they were aged 18 years or older, had a landline or cellular telephone with a USVI area code, were USVI residents, and were living in private or college housing. BRFSS collects information on individual demographics such as sex, age, and health outcomes, including diabetes diagnosis. BRFSS data were scaled to represent the adult population size by multiplying the BRFSS-provided sample weight by the ratio of the number of BRFSS observations divided by the sample weight sum.

We estimated subdistrict-level diabetes prevalence using the multilevel regression and poststratification approach (4,5). In stage 1, we estimated individual-level probability of diabetes using 2 models. In the first model, we obtained parameter estimates (ie, model coefficients) by using a multilevel logistic regression of 2021–2022 BRFSS data. This included individual-level (age and sex) and district-level (proportion of the population identifying as Black, Afro-Caribbean, or persons of color and proportion of the population living below 100% of the federal poverty level) fixed effects. The second model used the parameter estimates obtained in the first model to predict subdistrict-level prevalence by regressing counts of subdistrict-level population characteristics using 2020 US Decennial Census data. In stage 2, we used poststratification to produce model-based subdistrict-level estimates of diabetes prevalence. This process involved summing stage 1 individual-level expected probabilities over the individual-level population characteristic groups weighted by size of the subdistrict population.

We evaluated the model fit by examining prevalence estimate differences (territory-level direct BRFSS survey and model-based) and the mean square error (MSE) of district-level estimates. MSE is the sum of the squared difference in direct and model-based district-level estimates, divided by the number of districts.

We described geographic variation in subdistrict-level diabetes prevalence by mapping quintiles of diabetes prevalence. Analysis and mapping were conducted using R version 4.1.2 (6). This activity was reviewed by Centers for Disease Control and Prevention (CDC) and was conducted consistent with applicable federal law and CDC policy.

## Highlights

In the 2021–2022 BRFSS, 437 of 2,706 people (16%) responded that they had ever been told by a health care provider that they have diabetes. High concordance was reported between USVI direct BRFSS survey and model-based diabetes prevalence estimates (15% vs 16%) and low MSE (1.7), indicating acceptable model fit.

The map shows within-district heterogeneity in model-estimated prevalence, consistent with MAUP (3). St. Croix diabetes prevalence was estimated as 16% (subdistrict range, 6%–21%; interquartile range [IQR]: 9%). St. Thomas and Water Island prevalence was estimated as 17% (subdistrict range, 3%–28%; IQR, 7%), and St. John prevalence was estimated as 9% (subdistrict range, 3%–11%; IQR, 5%). The highest model-based prevalence estimates were for West End (28%; 95% CI, 14%–47%) and Tutu (26%; 95% CI, 16%–38%) subdistricts, both on St. Thomas.

## Action

This is the first analysis to estimate BRFSS chronic disease prevalence in USVI at a geographic unit smaller than the district level. Using 1 estimate for an entire district obscures local heterogeneity that might be relevant for understanding the drivers of higher diabetes prevalence and identifying localities for prioritizing public health programs. This was exemplified by Water Island’s estimate (3%) which was obscured by the St. Thomas and Water Island district-level estimate (17%). West End and Tutu subdistricts will be the first areas prioritized in diabetes program planning and outreach.

A limitation of our study was prevalence estimate imprecision, given the survey sample size for a limited population. Despite the large CIs, the estimates provide key information through the subdistrict ranked order to guide prioritization.

SAE was a valuable tool for prioritizing USVI subdistricts for diabetes public health interventions. Our analysis has implications beyond this investigation, serving as a proof-of-concept of SAE for other health measures.
